# Application of the Vienna Test System to Measure Training-Induced Changes in Choice Reaction Time in U20 Fencers: A 12-Week Training Program Pilot Study

**DOI:** 10.3390/sports13110400

**Published:** 2025-11-07

**Authors:** Lukass Edmunds Teteris, Sergejs Saulite, Renars Licis, Mara Greve, Behnam Boobani

**Affiliations:** 1 RSU Latvian Academy of Sport Education, Riga Stradins University, LV-1006 Riga, Latvia; lukass16@inbox.lv (L.E.T.); sergejs.saulite@rsu.lv (S.S.); renars.licis@rsu.lv (R.L.); 2 Statistics Unit, Riga Stradins University, LV-1007 Riga, Latvia; mara.greve@rsu.lv

**Keywords:** young athletes, fencing, training methods, choice reaction time, performance

## Abstract

This pilot study examined the effects of a 12-week reaction training program on physical and cognitive performance (choice reaction) in U20 Latvian fencers. Five qualified right-handed male fencers (aged 14.8–18.6 years) completed the Vienna Test System choice reaction task at baseline and after 12 weeks while cycling through five heart rate zones (1–5). Reaction speed (RS), motor speed (MS), choice reaction time (CR), and heart rate (HR) were recorded. Paired-sample *t*-tests indicated no significant group-level changes: RS (t = 1.46, *p* = 0.21, d = 0.65, 95% CI [−36.92, 118.92]), MS (t = 2.37, *p* = 0.07, d = 1.06, 95% CI [−3.14, 40.34]), CR (t = 1.70, *p* = 0.16, d = 0.76, 95% CI [−37.30, 156.26]), and HR (t = −2.69, *p* = 0.054, d = −1.20, 95% CI [−12.53, 0.17]). Individual responses revealed that three athletes improved CR in low- to moderate-intensity zones (−12.66% to −27.18%), whereas heart rate increased modestly (1.35% to 9.60%). Given the critical age for developing choice reaction, these findings should be considered as preliminary and exploratory, offering initial insights into how training might influence cognitive performance in young fencers and demonstrating that responses can differ across heart rate zones and among individuals.

## 1. Introduction

Fencing, which involves attacking and defending using a sword, is included in the Olympic Games [1]. It is a combat sport that demands open-skill strategies, complex body movements, and tactical play. Athletic performance in fencing reflects how effectively an athlete can execute actions. Like other sports, success relies on multiple physical and mental factors, including reaction time, agility, flexibility, coordination, and decision-making skills [2,3]. Fencing specifically requires athletes to respond quickly to tactile, visual, and auditory stimuli, which are the primary types of stimuli in fencing [4,5]. The blade, considered the second fastest object in sports competition, demands extremely rapid reactions. Typically, scoring actions last about 5 s in foil and 15 s in épée [6]. Therefore, responding effectively is an essential skill and performance [7,8].

Reaction time (RT) is a fundamental skill influencing success in fencing [9]. Reacting to a moving object involves several steps: perceiving the stimulus, estimating its speed, adopting an appropriate posture, and making a decision. In situations where multiple responses are possible, athletes rely on choice reaction time, which requires selecting the correct action under time pressure. This advantage is linked to shorter motor response times, which contribute to superior physical performance and, ultimately, better competitive results [9]. Furthermore, RT acts as an indicator of the efficiency of the cognitive system in processing information [10]. Cognitive processes are usually evaluated through behavioral measures like reaction time.

Cognitive skills and decision-making are essential for athletic performance in competitive sports, especially in combat sports like fencing [11,12]. In fencing, athletes need to constantly anticipate their opponent’s moves by interpreting cues or patterns in their behavior [13,14]. Research indicates that physical exertion and exercise can impact both cognitive performance and reaction time, but individual responses differ [15,16,17,18]. Studies using computer-based tasks indicate no significant differences between groups, or that elite or well-trained fencers typically have faster reaction and choice reaction times than less experienced athletes [18,19].

Some studies indicate that reaction time (RT) in fencers can be improved through training [20,21,22]. Conversely, other research contends that genetic factors mainly influence RT and are less affected by training [23]. To anticipate attacks, respond, and stay accurate, fencers must extract relevant information from an opponent’s constantly changing posture, distance, movements, or even the current match status [13]. Therefore, fencing demands quick and accurate decision-making under ongoing competitive pressure, along with an evolving perception of physical effort 18]. Since athletes are always under time limitations, both decision-making and sensorimotor reaction times need to be minimized to increase performance [8]. Improving the fencing training system involves identifying and justifying new methods for organizing training, based on modern scientific evidence. The Vienna test system (VTS) [24] is a psychological assessment device that includes tests of many different constructs, such as reaction time and attention, used by applied practitioners to conduct psychological testing in fencing [19,24,25]. Physical preparation is a key part of fencing performance, including explosive starts, sustained readiness for the right moment to attack, and the ability to make quick decisions [26]. Reactions are a crucial part of performance and are closely related to cognitive performance in fencers [27]. Several studies highlight the importance of improving reaction time and response speed to both auditory and visual stimuli, dual-task conditions, the role of speed in a fencer’s hand movements, and various training interventions [2,19,26,28,29,30,31,32]. This pilot study aimed to assess the effects of a 12-week reaction training program on physical and cognitive performance (choice reaction) in U20 Latvian fencers. We hypothesized that the targeted training program would improve choice reaction performance.

## 2. Materials and Methods

### 2.1. Participants

Five qualified young right-handed male foil fencers from the Latvian national U20 team, all ranked among the top athletes nationally, participated in the study (Table 1). The inclusion criteria included (a) age ranging from 14 to 20 years, which is a critical period for developing choice reaction [28], and (b) training at least 4 times per week. Exclusion criteria were (a) use of drugs, medications, or any conditions (neuromuscular diseases), or musculoskeletal injuries that could affect test outcomes, and (b) previous experience with the VTS. A qualified athlete in this study is defined as a fencer who is in the top 10 of the Latvian rankings, has participated in national and international competitions, and has an EFC (European Fencing Confederation, https://www.eurofencing.info/rankings/individual-rankings, URL accessed on 23 October 2025) or FIE (International Fencing Federation, https://fie.org/athletes, URL accessed on 23 October 2025) ranking. Participants ranged in age from 14.8 to 18.6 years (m = 17.04, SD = 1.43), with an average height of 179.2 cm (SD = 6.87) and body weight of 66.02 kg (SD = 10.33). The study followed the ethical regulations of the Declaration of Helsinki. Information about the purpose of the study was given to the participants, and they voluntarily consented to take part. For underage athletes, written informed consent was additionally obtained from parents or legal guardians. The research protocol was approved by the Ethical Committee of Riga Stradins University, Latvian Academy of Sport Education (protocol no. 8/3/51813, and approval date 30 July 2023). Participants could withdraw at any time without consequence.

### 2.2. Procedures

The experimental protocol included a baseline assessment (pre-test) and a follow-up assessment after 12 weeks (post-test). None of the participants had previous experience with the cognitive tasks of the Vienna choice reaction test. Therefore, before the intervention, all athletes completed two familiarization sessions, during which they practiced the choice reaction (CR) test while cycling for 30 min on a stationary bike [33]. This ensured that participants were familiar with the test procedures. One week later, the baseline assessment was conducted. At both pre- and post-test, participants performed the CR test five times while cycling on a cycle ergometer (Wattbike Pro/Trainer, Wattbike Ltd., Nottingham, UK) across five heart rate zones (Z1–Z5), monitored using a Polar heart rate system (Polar Team Pro, Polar Electro Oy, Kempele, Finland). The zones were defined according to a previous study [34] and Tanaka’s age-based prediction equations for maximal heart rate [208 − (age × 0.7)] [35]: zone 1 (50–59% HRmax), zone 2 (60–69% HRmax), zone 3 (70–79% HRmax), zone 4 (80–89% HRmax), and zone 5 (90–100% HRmax).

Upon arriving at the laboratory, participants sat and rested for 10 min. They first did a 10 min warm-up on the cycle ergometer. The protocol was performed in stages, with each stage corresponding to one heart rate zone. Each stage and the CR test lasts between 3 and 5 min. A total of five CR tests were performed. At the end of each stage, participants increased their pedaling intensity to reach the next target zone. During testing, the CR response panel was mounted on the ergometer. Workload progression was not standardized by resistance settings but was instead guided by heart rate response and self-selected pedaling intensity. The entire test took approximately 25–30 min.

Participants were advised to refrain from alcohol, caffeine, and vigorous physical activity for at least 24 h prior to testing. Participants were asked verbally to confirm they followed the pre-test instructions and reported full compliance. If they were noncompliant, testing would be rescheduled.

The Vienna Test System (VTS) is a popular computerized tool used for objectively assessing various psychological constructs. It is commonly employed in sports science to examine how different factors influence cognitive performance [24]. During the CR test, stimuli include yellow and red circles, auditory tones, and combinations of these. The key stimulus combination requires participants to respond when both a yellow and a red circle appear simultaneously. Two output variables from the CR test were recorded. Reaction speed (RS) is the time elapsed from when the relevant stimulus appears to when the participant’s finger lifts from the rest position button. It indicates how quickly an individual perceives a stimulus and initiates a response. Motor speed (MS) represents the second component of CR and is defined as the interval from when the finger lifts off the rest button to when the designated response is pressed. MS provides an index of processing and motor execution speed. The CR test lasts between 3 and 5 min. All procedures were non-invasive and supervised by the researcher and fencing coach.

### 2.3. Intervention

The modified reaction training program [21,36] was conducted twice weekly for 12 weeks (July–September 2024), with each session lasting 30 min and including 10 exercises (Appendix A, Table A1, Table A2 and Table A3). This program was performed alongside participants’ regular fencing and general training. The training protocol involved both general and fencing drills and was adapted from different sports to improve reaction time and cognitive performance [Table 2]. Each general and regular fencing session, without emphasizing reaction exercises, began with a standardized warm-up and continued with stretching, jogging, footwork, and tactical and technical training. Special training consisted of 10 reaction exercises using various equipment, including balls of different sizes and weights, tennis balls, jump ropes, and agility ladders. Exercises included catching balls, performing single-leg hops, cone-color reaction tasks, fencing drills, and combined movement and cognitive challenges such as jumping while catching a glove and shuttle runs. Sessions ended with cool-down exercises. The first author and a fencing coach oversaw all training sessions. Every fencer participated in each session.

### 2.4. Statistical Analysis

Data were analyzed using Microsoft Excel 2016 and Jeffreys’s Amazing Statistics Program (JASP) version 0.18.3. Due to the limited sample size and based on earlier research [14,37], descriptive statistics, including the minimum (Min), maximum (Max), mean (m), standard deviation (SD), effect sizes (Cohen’s d), confidence intervals (CIs), and percentage change (%Change), were calculated. Data normality was assessed using the Shapiro–Wilk test. A paired *t*-test (t) was used for data analysis. The significance level was *p* < 0.05. Percentage change (%Change) was calculated using the following formula:

%Change = [(mpost − mpre)/mpre] * × 100. (1)

## 3. Results

The results are divided into two parts: (1) overall pre–post changes in reaction speed (RS), motor speed (MS), choice reaction (CR), and heart rate (HR), and (2) individual zone-based profiles showing within-athlete changes over the 12-week training period. For RS, MS, and CR, lower values and reduction (milliseconds, ms) indicate faster responses, while HR values are given as average beats per minute (bpm).

### 3.1. Overall Pre–Post Results of RS, MS, CR, and HR

Reaction Speed (RS). Descriptive statistics for RS across participants are presented in Table 3. Overall post-test results showed a decrease in RS for S1, S3, and S5, indicating faster responses. The most improvement was seen in S1, with RS decreasing from m = 560.4, SD = 63.85 ms at pre-test to m = 415.4, SD = 27.55 ms at post-test, a −25.87% change. S3 and S5 showed moderate improvements of −12.45% and −8.67%, respectively. Conversely, S2 exhibited an increase in RS (3.49%), indicating slower responses, while S4 remained unchanged.

The motor speed (MS). Table 4 shows the results of MS, which demonstrated consistent improvements in four athletes. S1 indicated the greatest change, decreasing from m = 127.4, SD = 21.36 ms to m = 85.40, SD = 17.53 ms, reflecting a −32.96% and a large reduction. Notable decreases were also observed in S5 (−26.64%), S3 (−17.14%), and S2 (−15.36%). S4 showed an increase (4.85%).

Choice reaction (CR) and heart rate (HR). CR followed a similar trend (Table 5), with S1 decreasing substantially (m = 687.8, SD = 76.02 ms to m = 500.8, SD = 38.80 ms), resulting in a −27.18% reduction in post-test. The HR was increased from m = 124.9, SD = 26.4 to m = 141.4, SD = 28.5 bpm (8.85%). CR improvements were also evident in S3 (−13.37%), with improvements in HR (9.6%) and S5 (−12.66%), as well as HR (2.39%) in the post-test. Conversely, CR in S2 (0.53%), with a HR (1.35%), and S4 (1.25%), HR (1.5%) showed negligible increases.

Group level. Paired-sample *t*-tests (Table 6) examined pre- and post-test differences in RS, MS, CR, and HR. Shapiro–Wilk tests indicated normal distributions for all variables (*p* > 0.05). No significant changes were observed for RS, t(4) = 1.46, *p* = 0.218, d = 0.65, or CR, t(4) = 1.70, *p* = 0.163, d = 0.76. However, effect sizes indicate moderate changes in cognitive-motor performance. MS showed a trend toward improvement, t(4) = 2.37, *p* = 0.076, d = 1.06, and HR approached significance, t(4) = −2.69, *p* = 0.054, d = −1.20.

### 3.2. Individual Zone-Based Profiles of Athletes

**Figure 1:**
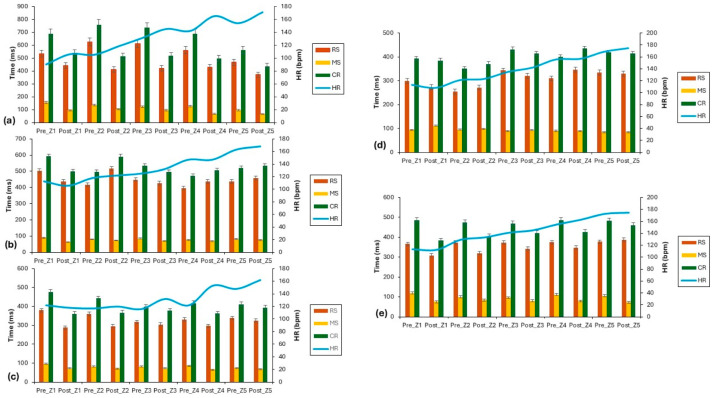
Individual zone profiles per athlete (*n* = 5); subfigures (**a**–**e**) correspond to subjects 1–5, respectively.

The results of individual zones are shown in Figure 1. Subject 1 showed consistent and substantial cognitive performance improvements across all zones (Figure 1a). RS decreased by −17% to −34% across Z1–Z5, with the largest in Z2 (−34%). MS is dramatically affected, especially in Z4 (−47.6%) and Z1 (−38.7%). Correspondingly, CR decreased between −21.9% and −32.4%. However, HR responses increased by 10–18% across all zones. S2’s profile (Figure 1b) was more mixed. RS was reduced in Z1 (−13%) and Z3 (−4.7%), but increased in Z2 (24.8%) and Z4 (10%). MS consistently improved (−7% to −30%), with the greatest change in Z1. Results for CR show a reduction in Z1 (−15.7%) and Z3 (−6.9%) but an increase in Z2 (19.4%) and Z4 (7%). HR responses were stable to slightly elevated (−6% to 5.6%).

S3 showed a strong and generalized reduction across most zones (Figure 1c). RS decreased by −24% in Z1, −17.8% in Z2, and around −4% in Z3–Z5. MS shows a reduction in all zones (−8% to −25%), with the best in Z1. CR decreased by −24.5% in Z1, −17% in Z2, and moderately in Z3–Z5. HR patterns, however, indicated modest decreases in Z1 (−3.3%) but increases up to 25% in Z4. The results for S4 show the most variable profile (Figure 1d). RS decreased in Z1 (−8.7%) and Z3 (−6.4%), but increased in Z2 (6.7%) and Z4 (11.6%). MS increased in Z1 (19.1%) and Z2 (4.2%), and stability was maintained in Z5. CR showed small changes (−2% to 8.8%). HR increased modestly across most zones (0.6% to 5.2%). The results of RS for S5 show a reduction in Z1–Z4 (−7% to −16%), but Z5 increased by 2.6%. MS decreased across all zones (−16% to −37.5%), with the largest reduction in Z1. CR also decreased across the zones (−4.7% to −21.3%). HR changes were minimal (−0.9% to 5.2%).

## 4. Discussion

This study examined the effects of a 12-week reaction training program on physical and cognitive performance in U20 fencers. The main findings indicate that at the group level, paired-samples *t*-tests for reaction speed (RS), motor speed (MS), choice reaction (CR), and heart rate (HR) did not reach statistical significance. Nevertheless, individual-level analyses revealed that choice reaction performance improved post-intervention in athletes S1, S3, and S5, while it decreased for athletes S2 and S4, particularly when tested at different heart rate zones, highlighting individual variability. Heart rate changes were modest across participants, indicating that improvements in choice reaction performance were not strongly associated with cardiovascular responses. These findings should be interpreted as preliminary and exploratory, providing initial insights into how training may influence cognitive performance in young fencers and illustrating that responses can vary across heart rate zones and among individuals.

The cognitive performance is influenced by variables such as training intensity, duration, and the task, interacting to create complex effects on performance outcomes [38]. McMorris [39] proposed that exercise influences cognition by activating the hypothalamic and sympathetic–adrenal systems, thereby increasing catecholamine release. These biochemical changes affect neural processes involved in attention and information processing, though the exact relationship between exercise intensity and cognitive performance remains complex and not fully understood. Specifically, post-intervention CR decreased across fencers (m = 500.8, SD = 38.8 to m = 419.4, SD = 28.97 ms) compared to baseline (m = 687.8, SD = 76.02 to m = 480.2, SD = 7.69 ms), corresponding to percentage changes ranging from 0.53% to −27.18%. These findings indicate that although individual results varied, the group results, including the lack of a significant effect (*p* > 0.05) due to sample size, all participants completed the 12-week reaction training without dropouts, indicating that this training protocol is feasible to implement and support in future, larger-scale studies. Concurrently, changes in heart rate responses varied among participants (Table 4), highlighting individual differences in physiological adaptation to the intervention.

Furthermore, individual optimal heart rate zones for achieving faster choice reaction times were observed in zones 1–2 (HR range: 106–118 bpm), with noticeable variation among participants (Figure 1). This result is consistent with earlier studies [40,41,42,43,44], which have shown that low to moderate intensity exercise improves response speed to choice reaction and visual stimuli. Specifically, the relationship between reaction time and exercise intensity seems to follow an exponential pattern: reaction time decreases as exercise intensity increases to the aerobic threshold (around 120 bpm), then plateaus between 130–155 bpm, and gradually rises again beyond the anaerobic threshold, continuing up to maximal effort. Stimuli further influence the impact of higher intensities (above 160 bpm).

In contrast to our findings, some studies have shown that low-intensity exercise does not significantly influence cognitive task performance, while higher intensities may improve reaction speed [15,16,17,18]. This partially aligns with previous research suggesting a U-shaped relationship of training intensity and cognitive performance, where moderate exercise has a beneficial effect but high-intensity training may impair performance [45]. However, the inverted-U model has faced criticism, and newer studies highlight that the impact of training on cognitive performance is related to the task [35]. Furthermore, athletes and non-athletes respond differently to exercise. Several studies comparing experienced athletes with novices indicate that athletes tend to benefit more from exercise, likely because of their greater familiarity with high physiological stress and the substantial physical, emotional, and cognitive demands involved [46,47,48,49].

In this study, we also investigated the effect of a 12-week training program on heart rate (HR). The increase was modest, ranging from 1.35% to 9.60% across participants, with the largest increase observed in S3 (9.60%) and the smallest in S2 (1.35%). Exercise is known to trigger both immediate and long-term adaptations in HR [50]; however, the relatively low training load of the current program may explain the limited HR changes in some athletes. Although sessions were held twice weekly for 30 min over 12 weeks, the ten exercises included were not enough to produce significant cardiovascular adaptation. Endurance training causes significant changes in cardio-autonomic regulation [51], which were not achieved in the current intervention. Importantly, improvements in CR performance observed in S1, S3, and S5 occurred despite modestly elevated HRs. This aligns with dual-task paradigms [33,44], where athletes must maintain cognitive performance while performing physical tasks, reflecting adaptations relevant to the training and fencing competition. Previous research has also shown that fatigue caused by high-intensity exercise generally leads to increased and longer reaction times [41].

Task complexity is another key factor affecting how exercise influences cognitive performance. Cognitive load results from the interaction between exercise, brain catecholamines, which increase arousal by activating the reticular formation, and the cognitive task. In this study, athletes S2 and S4 showed increased CR, aligning with previous findings [38]. A likely reason is that the combination of pedaling for 30 min and performing the test created a higher cognitive and physical load, leading to longer reaction times. Conversely, other fencers (S1, S3, and S5) completed these complex tasks with shorter CRs. The longer CRs seen in S2 and S4 may be related to the wait-and-see strategy [52], where additional cognitive processes like decision-making and information integration extend reaction time. Therefore, the amount and type of information needed to generate a response depend on the task and help explain individual differences in CR performance [53]. Moreover, caution is advised when comparing results from different studies, especially when the CR or experimental setup varies [54]. In this study, we used the CR test on a bicycle, a new laboratory measure for this group, since fencers usually do not use the VTS in their regular training. The familiarization was limited to 2 sessions, which may explain differences in cognitive performance. This could make measurements reflect the effect of being new to an unfamiliar dual task, where cycling demands much cognitive capacity and could interfere with other tasks (VTS), rather than measuring true cognitive impact.

Attention, which depends on executive functions like inhibitory control and working memory, also affects choice reaction time and overall performance [55,56]. In fencing, cues can appear anywhere in the visual field, requiring athletes to use peripheral vision and quickly move their gaze through rapid eye movements. Previous research shows that elite fencers are generally better at focusing on these cues [14]. In this study, two athletes (S2 and S4) showed longer choice reaction times, possibly indicating reduced or distracted attention, which can hinder information processing and fencing performance. Importantly, the critical age for developing CR is between approximately 15 and 20 years old [28]. In this study, the fencers were in a crucial age period when differences in reaction time and related variables are most likely to be detected. However, it is important to recognize that multiple factors, including age, ambient temperature, type of stimulus, nerve conduction quality, and receptor sensitivity, influence individual differences in outcomes. While the findings partly support the hypothesis that 12 weeks of training could improve choice reaction, there are several limitations. First, the small sample size of five males was determined by participant availability and by the inclusion and exclusion criteria of the study during data collection, rather than by predefined sample size calculations. Especially considering the critical age for developing choice reaction [28] and the fact that athletes (under 20 years old) and competing at the national or international level. Although they represent a rare and highly selective group, this complicates the recruitment of larger cohorts. Additionally, this study’s limitations include the lack of a control group and the absence of randomization. Future studies should increase the number of participants (including left-handed fencers) and add other specific weapons (such as épée and saber), which would be beneficial. Future research with larger sample sizes and longer data collection periods could provide more solid insights into the effects observed in this study. Second, the results obtained in a controlled laboratory setting may not directly apply to field environments [57] or actual competitions, as the procedures used here do not consider key factors in fencing, such as dynamic interaction with an opponent [58]. While this study focused on cognitive performance, which is crucial for executing fencing skills, a full understanding of how physical load influences cognitive expertise requires examining the underlying neurophysiological mechanisms. The present study included only male foil fencers aged 14–18. Future studies should expand the sample to include female athletes, athletes of different ages, and various levels of expertise. Because of differences in segment length and muscle mass, sex-related variations in execution speed could also be worth exploring. Since competitive fencing has been linked to cognitive fatigue, examining how it affects execution speed and accuracy could be valuable. Additionally, since the 12-week training program was tested with a small group, adding a control group would help provide clearer conclusions about its effect on cognitive performance, especially choice reaction. Results cannot establish causality. Observed changes may reflect learning or familiarization.

## 5. Conclusions

The present pilot study examined the effects of a 12-week reaction training program on physical and cognitive performance (choice reaction) in U20 Latvian fencers. While the group results showed no significant changes in the variables, some individual changes suggested that a 12-week training program may improve choice reaction time. Also, the current program’s low training load may explain the limited HR changes in some athletes. Given the critical age for developing choice reaction, a small sample size may limit the study, and these findings should be interpreted as preliminary and exploratory, providing initial insights into how training may influence cognitive performance in young fencers and illustrating that responses can vary across heart rate zones and among individuals. However, it is important to recognize that multiple factors, including age and stimulus type, influence individual differences in outcomes. These points emphasize the need for future research with highly controlled designs, including clear definitions of participant expertise, specific exercise protocols, and well-defined cognitive tasks.

## Figures and Tables

**Table 1:** Individual characteristics and ranking of fencers.

Subject	Age (Years)	Height (cm)	Body Mass (kg)	HR Rest (bpm)	EFC Rank *	FIE Rank *
S1	17.9	186	60	62	-	47
S2	17.1	178	62	79	328	-
S3	18.6	186	82.1	85	-	477
S4	16.8	170	56	76	48	549
S5	14.8	176	70	76	469	-

* cm: centimeter; kg: kilogram; bpm: beat per minute. * Ranking for the years 2023–2034; EFC: European Fencing Confederation; FIE: International Fencing Federation.

**Table 2:** The weekly training schedule.

Day	Monday	Tuesday	Wednesday	Thursday	Friday
Trainings	General training (60 min)	General (30 min) + special training (30 min)	Rest	General (30 min) + special training (30 min)	General training (60 min)

**Table 3:** Overall RS results (*n* = 5).

Variable	Sub	Test Phase	m ± SD	95% CI Lower, Upper	Min	Max	%Change
RS (ms)	S1	Pre	560.4 ± 63.85	481.11, 639.69	467	625	−25.87
		Post	415.4 ± 27.55	381.18, 449.61	371	444	
	S2	Pre	440.6 ± 40.78	389.96, 491.23	397	504	3.49
		Post	456 ± 36.47	410.70, 501.29	428	518	
	S3	Pre	345.2 ± 24.89	314.29, 376.11	317	380	−12.45
		Post	302.2 ± 14.09	284.69, 319.70	288	325	
	S4	Pre	308.4 ± 34.81	265.17, 351.62	255	343	0
		Post	308.4 ± 33.97	266.21, 350.58	272	346	
	S5	Pre	373.6 ± 4.03	368.58, 378.61	367	378	−8.67
		Post	341.2 ± 30.77	302.98, 379.41	308	388	

RS: reaction speed; ms: milliseconds; Sub subject; m mean, SD: standard deviation; CI: confidence interval; Min: minimum, Max: maximum.

**Table 4:** Overall MS results (*n* = 5).

Variable	Sub	Test Phase	m ± SD	95% CI Lower, Upper	Min	Max	%Change
MS (ms)	S1	Pre	127.4 ± 21.36	100.87, 153.92	96	155	−32.96
		Post	85.40 ± 17.53	63.63, 107.16	66	103	
	S2	Pre	82 ± 5.29	75.43, 88.57	75	89	−15.36
		Post	69.4 ± 5.03	63.15, 75.64	62	75	
	S3	Pre	84 ± 8.45	73.50, 94.49	74	97	−17.14
		Post	69.6 ± 3.05	65.81, 73.38	65	72	
	S4	Pre	90.6 ± 4.03	85.58, 95.61	85	95	4.85
		Post	95 ± 10.52	82.53, 108.67	85	112	
	S5	Pre	106.6 ± 9.42	94.89, 118.30	96	120	−26.64
		Post	78.2 ± 4.32	72.83, 83.56	73	84	

MS: motor speed; ms: milliseconds; Sub: subject; m mean, SD: standard deviation; CI: confidence interval; Min: minimum, Max: maximum.

**Table 5:** Overall CR and HR results in pre- and post-tests (*n* = 5).

Variable	Sub	Test Phase	m ± SD	95% CI Lower, Upper	Min	Max	%Change
CR (ms)	S1	Pre	687.8 ± 76.02	593.39, 782.20	563	760	−27.18
		Post	500.8 ± 38.8	452.61, 548.98	437	539	
	S2	Pre	522.6 ± 45.88	465.62, 579.57	472	593	0.53
		Post	525.4 ± 39.51	476.33, 574.46	497	591	
	S3	Pre	429.2 ± 31.09	390.59, 467.80	398	477	−13.37
		Post	371.8 ± 13.55	354.97, 388.62	360	393	
	S4	Pre	399 ± 31.49	359.89, 438.10	350	432	1.25
		Post	404 ± 25.65	327.14, 435.85	371	435	
	S5	Pre	480.2 ± 7.69	470.64, 489.75	470	487	−12.66
		Post	419.4 ± 28.97	383.42, 455.37	383	461	
HR (bpm)	S1	Pre	124.93 ± 26.4	92.14, 157.72	90	154	8.85
		Post	141.43 ± 28.53	105.99, 176.86	106	171	
	S2	Pre	133.2 ± 21.12	106.97, 159.42	113	163	1.35
		Post	135 ± 23.72	105.53, 164.46	106	168	
	S3	Pre	125 ± 13.15	108.66, 141.33	116	148	9.6
		Post	137 ± 19.72	112.51, 161.48	118	162	
	S4	Pre	138.8 ± 23.47	109.64, 167.95	113	169	1.5
		Post	(141 ± 26.58)	107.99, 174	108	175	
	S5	Pre	142.2 ± 23.13	113.47, 170.92	113	173	2.39
		Post	145.6 ± 24.77	114.83, 176.36	112	175	

CR: choice reaction; ms: milliseconds; HR: heart rate; bpm: beats per minute; Sub: subject; m mean, SD: standard deviation; CI: confidence interval; Min: minimum, Max: maximum.

**Table 6:** *t*-test results for RS, MS, CR, and HR.

Sub	Test Phase	m ± SD	95% CI Lower, Upper	t	*p*	d
RS (ms)	Pre	405.64± 99.13	−36.92, 118.92	1.46	0.218	0.65
	Post	364.64 ± 68.07				
MS (ms)	Pre	98.12 ± 19	−3.14, 40.34	2.37	0.076	1.06
	Post	79.52 ± 10.91				
CR (ms)	Pre	503.76 ± 113.26	−37.30, 156.26	1.70	0.163	0.76
	Post	444.28 ± 65.7				
HR (bpm)	Pre	133.82 ± 6.86	−12.53, 0.17	−2.69	0.054	−1.20
	Post	140 ± 4.13				

Sub: subject; RS: reaction speed; MS: motor speed; CR: choice reaction; HR: heart rate; ms: milliseconds; bpm: beats per minute; m: mean; SD: standard deviation; CI: confidence interval; t: *t*-test; *p*: *p*-value < 0.05; d: Cohen’s effect size.

## Data Availability

Data are available upon request to the correspondence author. An anonymized dataset/code will be open access (for reproducibility).

## Abbreviations

The following abbreviations are used in this manuscript:

RT	Reaction time
VTS	Vienna test system
CR	Choice reaction
RS	Reaction speed
MS	Motor speed
m	mean
SD	Standard deviation
%Change	Percentage change
ms	milliseconds
Sub	subject
Min	minimum
Max	maximum
HR	Heart rate
bpm	beats per minute
Z	Zone

## Appendix A

**Table A1:** The 12-week training program description (2× a week), month 1.

No.	Instructions	Dosage
1	Launch a tennis ball, let it bounce, and catch it with one hand while extending your arm (from the guard position)	2 × 1 min + rest 30 s
2	Throw a reaction ball and grab it with one hand after it hits the ground.	2 × 1 min + rest 30 s
3	In the same exercise, catch the ball during the drill, then immediately perform a forward lunge.	2 × 1 min + rest 30 s
4	Agility leather exercises: step forward, then move your right foot outside the square boundary.	2 × 1 min + rest 30 s
5	Agility leather exercises: step ahead, then shift your left foot beyond the edge of the box.	2 × 1 min + rest 30 s
6	Begin in a defensive stance, take two strides forward, and one step back.	2 × 1 min + rest 30 s
7	Start from the guard position, walk to the first marker, then retreat to the next, repeat this relay-style movement.	2 × 1 min + rest 30 s
8	Practice fencing footwork from a ready stance, changing direction whenever the coach claps.	2 × 1 min + rest 30 s
9	Moving into the fighting stance, the individual responds to cones of various colors.	2 × 1 min + rest 30 s
10	Jogging in place in a fencing stance and striking the fencing target on a coach’s command.	2 × 1 min + rest 30 s

**Table A2:** The 12-week training program description (2× a week), month 2.

No.	Instructions	Dosage
1	Throw the tennis ball up, catch it smoothly, and stretch your arm forward quickly.	2 × 1 min + rest 30 s
2	Put two colored objects in front. When the coach says a color, touch the one that matches.	2 × 1 min + rest 30 s
3	Two athletes stand apart 2 m and step forward to tap the object in front of them.	2 × 1 min + rest 30 s
4	Each athlete has two colored items. When the coach gives the signal, they race to grab the right one (reaction play).	2 × 1 min + rest 30 s
5	Agility leather exercises: Jump into each box using only the right foot.	2 × 1 min + rest 30 s
6	Agility leather exercises: jump onto each box using only the left leg.	2 × 1 min + rest 30 s
7	From the guard position, when told, take two fast steps forward and wait for the next signal.	2 × 1 min + rest 30 s
8	Move around freely. When signaled, lunge forward, go back to ready stance, take two quick steps back, and repeat	2 × 1 min + rest 30 s
9	Running in place in the fencing stance and catching the fencing glove with a lunge.	2 × 1 min + rest 30 s
10	Jumping rope and catching the fencing gloves.	2 × 1 min + rest 30 s

**Table A3:** The 12-week training program description (2× a week), month 3.

No.	Instructions	Dosage
1	Place two colored posts in front. When the coach says a color, the athlete quickly taps that one using their strong hand.	2 × 1 min + rest 30 s
2	Two athletes stand facing each other with a post between them. When the coach gives the signal, they race to grab it—whoever gets it first wins.	2 × 1 min + rest 30 s
3	Exercise no. 2 is repeated, and the coach shouts different commands: hand on knee, hand on head, etc. When he shouts and puts his hand on the pole, the athletes must quickly grab the pole.	2 × 1 min + rest 30 s
4	While jumping rope, reach out and catch the fencing glove during the movement.	2 × 1 min + rest 30 s
5	Step forward and backward like in fencing. When signaled, sprint in place with high knees from the guard position.	2 × 1 min + rest 30 s
6	Do jumping jacks, then catch the fencing glove when prompted.	2 × 1 min + rest 30 s
7	Run back and forth between two points, and catch tennis balls during the drill (Shuttle running).	2 × 1 min + rest 30 s
8	Move freely. When the coach signals, take two fast steps back, then lunge forward with power.	2 × 1 min + rest 30 s
9	Jump from a distant spot toward a closer one using agility leather.	2 × 1 min + rest 30 s
10	Make two forward jumps with both feet, then jump backward once.	2 × 1 min + rest 30 s
